# Editorial: Nutritional support in pediatric cancer: novel insights and future perspectives

**DOI:** 10.3389/fnut.2024.1397439

**Published:** 2024-05-02

**Authors:** Edoardo Muratore, Davide Leardini, Francesco Baccelli, Francesco Fabozzi

**Affiliations:** ^1^Pediatric Hematology and Oncology, IRCCS Azienda Ospedaliero-Universitaria di Bologna, Bologna, Italy; ^2^Department of Hematology/Oncology, Cell and Gene Therapy – IRCCS, Bambino Gesù Children's Hospital, Rome, Italy

**Keywords:** pediatric cancer, pediatric hematological diseases, nutrition, microbiome, nutritional support

Nutritional support is emerging as a crucial aspect, yet often overlooked in the treatment of pediatric oncology patients (1, 2). Children with cancer are at high risk of developing short-term and long-term nutritional problems related to their underlying disease and side effects of multimodal treatments (3). Adult literature is growing quite rapidly while children are often less analyzed. Indeed, children represent a specific population with different nutrition issues and needs. Moreover, growing evidence is showing how improving nutritional status could influence clinical outcomes, reducing treatment-related toxicities and overall mortality (4).

Despite the increased focus on nutritional evaluation and support, there is currently a lack of a systematic approach to this issue as pointed out by the recent survey conducted by the Associazione Italiana di Ematologia ed Oncologia Pediatrica (AIEOP) (5). Among the main reasons for the high variability in nutritional approach among pediatric oncological centers, participants in the survey underlined the need for further solid evidence (5). Within this Research Topic, contributing authors aimed to reduce this gap via new research or by summarizing key aspects of the evolving nutritional care in pediatric cancer patients.

One of the main issues is how to assess nutritional status. In particular, the use of serum biomarkers to better measure malnutrition is lagging in childhood cancer patients. Runco et al. analyzed for the first time in children serum concentrations of growth differentiation factor 15 (GDF15), a known non-specific marker in adults of oxidative stress, inflammation, and cachexia. GDF15 levels were higher at diagnosis and during treatment compared to healthy subjects but they did not find any association with anthropometric measurements or quality of life assessments. Despite not correlating with nutritional status, this pilot study opens up a role of GDF15 as a potential therapeutic target for childhood cancer cachexia.

LaLonde et al. focused instead on the delivery of nutritional support to pediatric allogeneic hematopoietic cell transplantation (allo-HCT). In particular, they analyzed a very important yet neglected part of nutritional care, which is the caregivers' ability to support their children nutritionally in this peculiar population and highlighted the need to support the ones struggling emotionally and economically to optimize nutritional support.

These works underline two key concepts in the future perspectives of nutritional support in pediatric oncology. The search for novel serum biomarkers of malnutrition is pivotal in nutritional assessment because traditional measures, such as body mass index, are often unreliable during cancer treatment as they can be influenced by the hydration status and do not discriminate between muscle and adipose tissue (3). On the other hand, caregiver's empowerment represents a necessary step toward the improvement of dietary regimes of their children, enabling to further refine nutritional support to modulate the gut microbiome (6) and avoid malnutrition (2), even during treatment complications (7).

The role of nutrition in mitigating many of the side effects of cancer treatment is gaining significant interest not only during intensive therapy but also for long-term survivors. Considering that over 85% of childhood cancer patients survive after 5 years of follow-up (8), diet is increasingly being considered a key feature to mitigate the most common adverse long-term health outcomes, such as cardiovascular disease (8). Feit et al. and Guida et al. provide two interesting reviews on the current landscape and innovative strategies for nutritional assessments and interventions among childhood cancer survivors. Despite the lack of solid evidence in this peculiar population, both papers highlight the need for standardized guidelines and further studies to apply new approaches. Among the most interesting future perspectives, the role of artificial intelligence in predicting the risk for malnutrition and as a personalized nutritional assistant should be surely explored.

In conclusion, a lot of work still needs to be done in the field of nutritional support in pediatric cancer patients. Future research questions should focus on the different time points of nutritional support: the status at diagnosis, the nutritional support during the therapies, and the long-term nutritional consequences. More data on how nutritional assessment should be performed could improve clinical evaluation and provide a stronger correlation with clinical outcomes. Therefore, if nutritional status can be considered an independent and modifiable prognostic factor, nutritional support can be used to potentially improve patients' survival and quality of life.

Shared recommendations for the management of nutritional needs in pediatric oncology could represent a common ground to start improving in this field, as recently performed by AIEOP (9). This type of shared effort could guide physicians in everyday practice and provide a framework for future research. Moreover, the gut microbiome is emerging as a potential modulator of anti-cancer therapy efficacy and toxicity (10–12), and nutrition could represent a cost- and risk-effective strategy to interact with the intestinal flora toward a protective configuration (13). Nutritional support has the potential to be a diamond in the rough in the treatment of pediatric cancer, and we are just starting to scratch the surface.

## Author contributions

EM: Conceptualization, Writing – original draft, Writing – review & editing. DL: Conceptualization, Writing – original draft, Writing – review & editing. FB: Conceptualization, Writing – original draft, Writing – review & editing. FF: Conceptualization, Writing – original draft, Writing – review & editing.
